# Socioeconomic status influences sex ratios in a Chinese rural population

**DOI:** 10.7717/peerj.3546

**Published:** 2017-06-30

**Authors:** Liqun Luo, Rui Ding, Xiali Gao, Jingjing Sun, Wei Zhao

**Affiliations:** 1 Department of Sociology, Central China Normal University, Wuhan, China; 2 Hubei Institute of Economic and Social Development, Central China Normal University, Wuhan, China

**Keywords:** Socioeconomic status, Offspring sex ratio, Trivers–Willard hypothesis, Peasants

## Abstract

According to the logic of the Trivers–Willard hypothesis, in a human population, if socioeconomic status is transmitted across generations to some extent, and if sons of high-status parents tend to have higher reproductive success than daughters, while daughters of low-status parents tend to have higher reproductive success than sons, then we should expect that offspring sex ratio is positively associated with socioeconomic status. This study examines whether the assumptions and prediction of this hypothesis apply to a rural population in northern China. Results show that (1) current family socioeconomic status is positively related to family head’s father’s socioeconomic status in around 1950, (2) low-status family heads have more grandchildren through their daughters than their sons, whereas high- or middle-status family heads have more grandchildren through sons, and (3) as family heads’ status increases, they tend to produce a higher offspring sex ratio. Therefore, the assumptions and prediction of the hypothesis are met in the study population. These results are discussed in reference to past studies on sex ratio manipulation among humans.

## Introduction

The Trivers–Willard hypothesis (TWH) (Trivers & Willard, 1973) predicts that for all sexually reproducing species including humans, natural selection favors those parents who bias the sex ratio of offspring produced according to their ability to invest in offspring. More specifically, parents in good condition will produce relatively more sons, whereas parents in poor condition will produce relatively more daughters. For humans, the above-mentioned “condition” may be operationalized as socioeconomic status.

The original TWH prediction is with offspring sex ratio at birth, as implies that some physiological mechanisms may adjust the sex ratio at conception or in utero. Later Trivers (2002) realized that an extended TWH prediction regarding parental sex preferences in humans was “perhaps the most striking advance” since Trivers & Willard (1973). According to the extended prediction, high-status parents tend to prefer sons to daughters, while low-status parents tend to prefer daughters. It follows that parents may adjust offspring sex ratios by conscious behaviors such as sex-selective abortion and differential treatment of sons and daughters. As both physiological mechanisms and behavioral mechanisms can influence offspring sex ratios, the ultimate results will be that the offspring sex ratio will be higher for high-status parents, and lower for low-status parents. It is this prediction regarding offspring sex ratio that we tested.

In their original paper, Trivers & Willard (1973) based the logic of the hypothesis on three assumptions. For the sake of simplicity, the three assumptions can be reduced to two, i.e., (a) the condition of parents during parental investment is positively correlated to the condition of their offspring at adulthood, and (b) reproductive success is much more strongly related to the condition of males than to that of females (i.e., good-condition males tend to outproduce good-condition females, whereas poor-condition females tend to outproduce poor-condition males). Obviously, a more direct result following from the two assumptions is that sons of good-condition parents tend to have better reproductive prospects than their sisters, while daughters of poor-condition parents tend to have better reproductive prospects. Furthermore, in its generalized form, the TWH is sometimes stated as that natural selection should favor parents who bias offspring sex ratio toward the sex with better reproductive prospects (Schindler et al., 2015).

Studies show that the first assumption of the TWH holds in a variety of non-human species (Hewison & Gaillard, 1999) as well as in humans (Erikson & Goldthorpe, 1992; Lin & Bian, 1991). Clutton-Brock, Albon & Guinness (1986)’s study of red deer is perhaps the only study that tests and confirms the applicability of the second TWH assumption in an animal species. As for humans, the second assumption should be valid in both traditional and contemporary societies, as the reproductive success of high-status men exceeds that of high-status women, while low-status women outproduce low-status men (Betzig, 2012; Hopcroft & Martin, 2014, 2016). Moreover, Bereczkei & Dunbar (1997) discovered that Hungarian Gypsies who had a lower status than Hungarians had more grandchildren through their daughters, but this difference was marginal and inconsistent in Hungarians. Since this finding is a logical result of the two assumptions of the TWH, their study actually is also a test of the assumptions in that study population.

Many studies reported that empirical tests of the TWH prediction regarding offspring sex ratio produced inconclusive results. However, according to a meta-analysis by Cameron (2004), mammalian sex-ratio studies that used measures of maternal condition around conception provide almost unanimous support for the prediction that good-condition mothers are more likely to give birth to sons than daughters. Because sex-ratio adjustment may take place around conception, condition around conception seems to be a more appropriate predictor of sex ratio.

As for humans, many studies in both historical and contemporary societies have documented adaptive variation in offspring sex ratio in line with the TWH. These studies used different measures of parental or maternal condition. These measures include ethnicity (Bereczkei & Dunbar, 1997), specialized socioeconomic index developed by American sociologists (Hopcroft, 2005; Hopcroft & Martin, 2014), and ownership of dwelling (Wallner, Fieder & Seidler, 2012), for indicating overall socioeconomic status; nutritional status (Gibson & Mace, 2003); third-party-reported overall socioeconomic status (Luo, Zhao & Weng, 2016); and wife rank in polygynous marriage for indicating economic resources (Pollet et al., 2009). In contrast, some other studies only found weak support for the TWH (Chacon-Puignau & Jaffe, 1996) or even no support (Zaldivar, Lizarralde & Beckerman, 1991; Kolk & Schnettler, 2016). In Zaldivar, Lizarralde & Beckerman (1991), family size and birth order were assumed to affect parental condition in some way, and thus were used to predict offspring sex ratio. Chacon-Puignau & Jaffe (1996) and Kolk & Schnettler (2016) used a variety of socioeconomic indicators such as parental education and income for measuring parental condition. As in non-human mammals (Cameron, 2004), better measures of maternal or parental condition might have shown stronger Trivers–Willard effects on offspring sex ratios (Cronk, 2007; Hopcroft & Martin, 2014). Besides, since the TWH relies on differentiation on a socioeconomic scale (Trivers & Willard, 1973), smaller socioeconomic inequalities in a study population means a smaller Trivers–Willard effect. Then the effect might be difficult to visualize, as in the case of Kolk & Schnettler (2016). This is somewhat like that we cannot see microbes with the naked eye. Finally, compared to historical studies and small-scale studies in contemporary societies, large-scale studies in contemporary societies are less likely to detect a Trivers–Willard effect (Cronk, 2007). In this article, large-scale studies mainly refer to those which have a large sample from a national population, while small-scale studies have a relatively small sample from some local population.

The current study tested the TWH using data collected in a contemporary Chinese rural population. First, we tested whether the first assumption of the TWH, relatively stable intergenerational transmission of socioeconomic status, held in the Chinese peasants. Specifically, we examined whether peasant family heads’ socioeconomic status was correlated with their fathers’ socioeconomic status in around 1950. The second TWH assumption, i.e., socioeconomic status affects reproductive success of males more than that of females, is unable to be tested directly by our data. Instead, we then examined whether the logical result (that sons of high-status parents tend to have higher reproductive success than daughters) applied to the peasants. Finally, we tested whether high-status peasants were more likely to have sons among their offspring, while low-status peasants were more likely to have daughters.

## Methods

This study was approved by the Ethics Committee of the School of Sociology and Social Work, Central China Normal University (Approval number: 2016001). Participants (informants) gave oral informed consent to take part in the study. The data were analyzed anonymously.

### Data collection

This study involved 12 neighboring villages in Shenqiu County, eastern Henan Province, and northern China. In each village, we mainly relied on two informants to obtain information about each family. One informant was a current village head or accountant in his forties or fifties, while the other was a retired village head or accountant in his sixties. They were paid, beforehand, around 30 US dollars as reward. Then they informed us of each family head’s education level, family socioeconomic status, father’s previous family class identity, and number of sons, daughters, sons’ offspring, and daughters’ offspring. The informants had lived there for decades and were well familiar with local families. In most cases, both informants in each village agreed on the personal data of family heads. In other cases, we allowed them to discuss for a while freely to settle the disagreement. We finally obtained information about 1,416 family heads.

Our data collection method is similar to that adopted by the American anthropologist Wolf (1995) in collecting data about Taiwanese women’s extramarital relations in 1967–1968 and 1971–1972.

### Coding of variables

The above-mentioned “family class identity” was assigned by the Chinese Communist Party to each rural family in around 1950 on the basis of economic status. Inheritable through the male line, it consisted of seven categories: landlord, rich peasant, upper middle peasant, middle middle peasant, lower middle peasant, poor peasant, and landless laborer. Family class identities represented the overall sociopolitical and economic hierarchy in rural China before 1950, with landlords and rich peasants at the top rung (Deng & Treiman, 1997; Sato & Li, 2008). In our pilot fieldwork, we noticed that some informants did not distinguish between different middle peasant identities and between poor peasant and landless laborer. Hence, in our formal interview, we only required that the informants report one of the following four class identities of each family head: poor peasant/landless laborer, middle peasant, rich peasant, and landlord, which were encoded as 1, 2, 3, and 4, respectively.

We calculated the sex ratio of all children per family head as follows: number of sons divided by number of all children. Socioeconomic status of a family head was encoded as 1 if he/she has a low socioeconomic status in the local population, as 2 if he/she has a middle socioeconomic status in the population, and as 3 if from a high socioeconomic status.

By subtracting each family head’s number of daughters’ children from number of sons’ children, we obtained a new variable named as number of sons’ children_minus_number of daughters’ children.

Sex of a family head was measured as male = 1 and female = 0, while age of a family head was coded as age in years.

### Data analysis

In testing whether there were relatively stable intergenerational transfers of socioeconomic status, that is, the first TWH assumption, we used each family head’s current family socioeconomic status as dependent variable. The explaining variables were those characteristics of a family head. They included family head’s sex, age, years of education, number of offspring, and father’s previous class identity. As the dependent variable was an ordinal variable and the data did not meet the proportional odds assumption for performing an ordered logit regression, we performed an ordered probit analysis.

The data regarding grandchildren did not conform to a normal distribution. Thus, to examine whether family heads tended to have more grandchildren through their sons as socioeconomic status rose, we conducted Wilcoxon signed ranks tests, a kind of nonparametric test, to compare sons’ children and daughters’ children by family heads’ socioeconomic status. Also, we performed an ordered probit regression of the variable number of sons’ children_minus_number of daughters’ children (the proportional odds assumption was not met). The independent variables characteristics of a family head included family head’s sex, age, years of education, number of offspring, family socioeconomic status, and father’s previous class identity.

The primary purpose of this study is to test the TWH prediction that high-status parents have more sons among their offspring. First, we assumed that older or lower-education peasants tended to be influenced by traditional son-preference culture (Li & Cooney, 1993) to a larger extent, and that they were more likely to abort a female fetus or abuse their young daughters. Thus, they were expected to have a higher offspring sex ratio. Second, because number of offspring usually was negatively associated with offspring sex ratio in human populations (Mace & Jordan, 2005), we assumed that there was also such an association in the study population. Finally, we expected that there was a positive association between family head’s family socioeconomic status/father’s class identity and offspring sex ratio, as this was found in another rural Chinese population (Luo, Zhao & Weng, 2016). Therefore, we used each family head’s offspring sex ratio as dependent variable, and family head’s age, years of education, number of offspring, family socioeconomic status, father’s previous class identity, and sex, as independent variables. Because the data did not meet the linearity assumption as well as the proportional odds assumption, we used each family head’s offspring sex ratio as an ordinal variable and performed an ordered probit regression of this variable. We also performed ordered probit regressions of family head’s offspring sex ratio for the separate parity levels ranging from parity 1 to parity 6. In this case, the variable number of offspring was removed from the probit model.

Before each probit regression analysis mentioned above, we also conducted an ordinary least squares regression using the same dependent and independent variables to examine values of tolerances. These tolerance values can be used to detect potential multicollinearity in a logistic or probit regression model (Menard, 2002). In fact, multicollinearity was not an issue in this study.

Along with each probit regression analysis, we also performed an ordinal logit regression using the same variables. The logit models showed essentially the same results (specific analysis results will not be reported below).

All statistical analyses were conducted using Stata 12.0.

## Results

Table 1 below gives descriptive statistics for the variables used in the analyses.

**Table 1:** Descriptive statistics for family heads.

Variable	Mean	SD	Min	Max
Sex (male = 1, *n* = 1,416)	0.89	0.31	0	1
Age (*n* = 1,416)	57.95	11.71	27	97
Years of education (*n* = 1,416)	6.03	2.45	0.00	16.00
Number of offspring (*n* = 1,416)	2.50	1.05	0.00	6
Current family socioeconomic status (*n* = 1,416)	2.11	0.54	1	3
Father’s former class identity (*n* = 1,416)	1.21	0.69	1	4
Offspring sex ratio (*n* = 1,369)	0.59	0.28	0.00	1.00
Number of sons’ children (*n* = 1,415)	1.75	2.03	0.00	14
Number of daughters’ children (*n* = 1,407)	1.40	1.90	0	14
Number of grandchildren (*n* = 1,407)	3.15	3.05	0	16

In the family heads, there were 1,267 males and 149 females. The number of family heads with a low, middle, and high family socioeconomic status were 137, 988, and 291, respectively. The number of family heads whose father’s former class identity was poor peasants/landless laborer, middle peasant, rich peasant, or landlord were 1,274, 33, 57, and 52, respectively.

### Were there intergenerational transfers of socioeconomic status?

The ordered probit model (Table 2) showed that there was a significantly positive relation between a family head’s father’s socioeconomic status in around 1950 (measured as father’s former class identity) and the family head’s current family socioeconomic status. The effect size for father’s former class identity in predicting current family socioeconomic status (Spearman rank correlation coefficient *r*_s_) is 0.067 (*n* = 1,416, *p* = 0.011). The results mean that socioeconomic status did transfer across generations to some extent in the past decades. Therefore, the first TWH assumption is met.

**Table 2:** Ordered probit parameter estimates, regression of each family head’s family socioeconomic status on independent variables.

Independent variables	Coef.	SE	*p*	95% Conf.	
				Lower limit	Upper limit
Sex (male = 1)	0.067	0.107	0.534	−0.144	0.277
Age	−0.009	0.003	0.013	−0.016	−0.002
Years of education	0.126	0.016	0.000	0.095	0.157
Number of offspring	0.220	0.034	0.000	0.153	0.288
Father’s former class identity	0.107	0.047	0.024	0.014	0.199

### Did low-status family heads have more grandchildren through their daughters than their sons, whereas high-status family heads show the opposite pattern?

**Figure 1:**
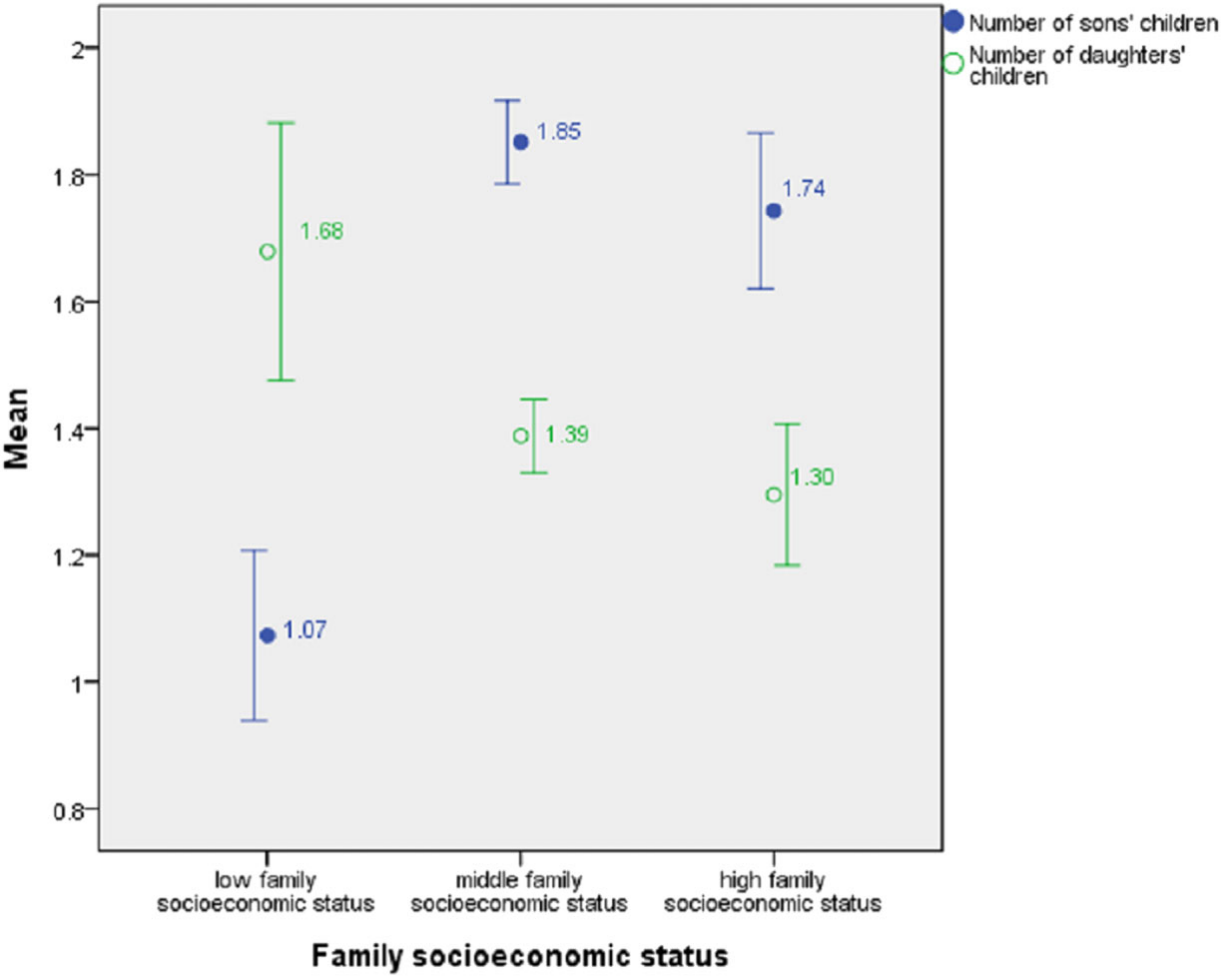
Number of sons’ children and daughters’ children by family socioeconomic status, showing mean ± 1 SE.

The data showed that in general, as family socioeconomic status declined, family heads tended to leave more grandchildren through their daughters than their sons (Fig. 1), consistent with the inference directly from the two TWH assumptions. We obtained the information about sons’ children of 137 low-status, 988 middle-status, and 290 high-status family heads, and the mean number of sons’ children for them were 1.07, 1.85, and 1.74, respectively. In contrast, for the 137 low-status, 982 middle-status, and 288 high-status family heads from whom we had data about daughters’ offspring, the mean number of daughters’ children were 1.68, 1.39, and 1.30, respectively. On average, low-status family heads left more grandchildren through their daughters (Wilcoxon signed ranks test, *p* = 0.005), whereas both middle-status and high-status family heads left more grandchildren through their sons (Wilcoxon signed ranks tests, *p* = 0.000 and *p* = 0.001).

The probit model (Table 3) showed that family head’s family socioeconomic status was positively correlated with the variable number of sons’ children_minus_number of daughters’ children when other variables such as family head’s age were controlled, that is, a higher status meant a larger excess of sons’ children over daughters’ children.

**Table 3:** Ordered probit parameter estimates, regression of each family head’s number of sons’ children_minus_number of daughters’ children on independent variables.

Independent variables	Coef.	SE	*p*	95% Conf.	
				Lower limit	Upper limit
Sex (male = 1)	0.136	0.092	0.139	−0.044	0.315
Age	0.019	0.003	0.000	0.013	0.025
Years of education	−0.037	0.013	0.006	−0.063	−0.011
Number of offspring	−0.200	0.030	0.000	−0.259	−0.141
Father’s former class identity	0.001	0.040	0.988	−0.077	0.079
Family socioeconomic status	0.283	0.053	0.000	0.179	0.387

The effect size (Spearman *r*_s_) between family socioeconomic status and number of sons’ children_minus_number of daughters’ children is 0.069 (*n* = 1,407, *p* = 0.009).

### Was family socioeconomic status positively correlated with offspring sex ratio?

**Figure 2:**
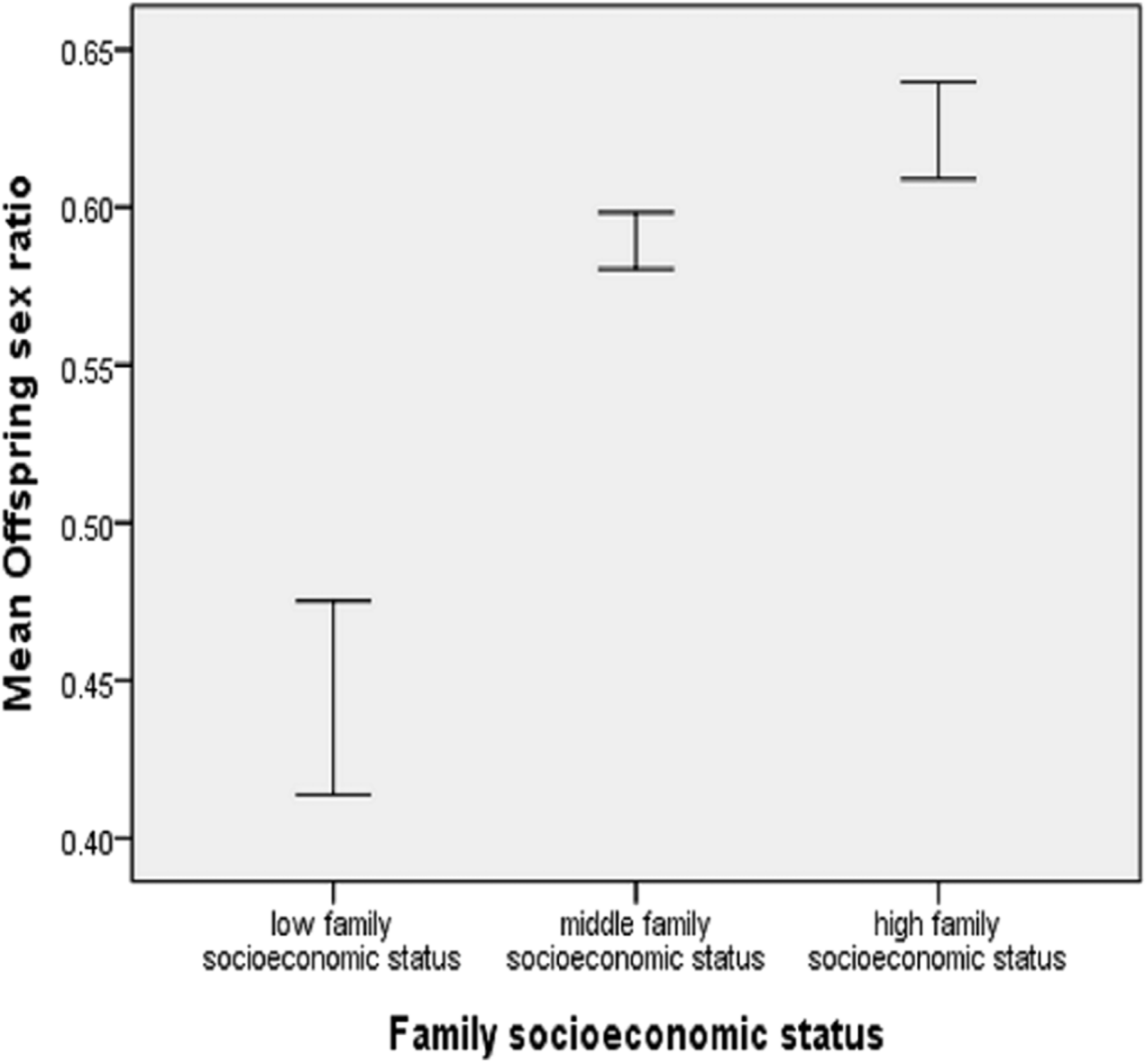
Offspring sex ratio by family socioeconomic status, showing mean ± 1 SE.

**Figure 3:**
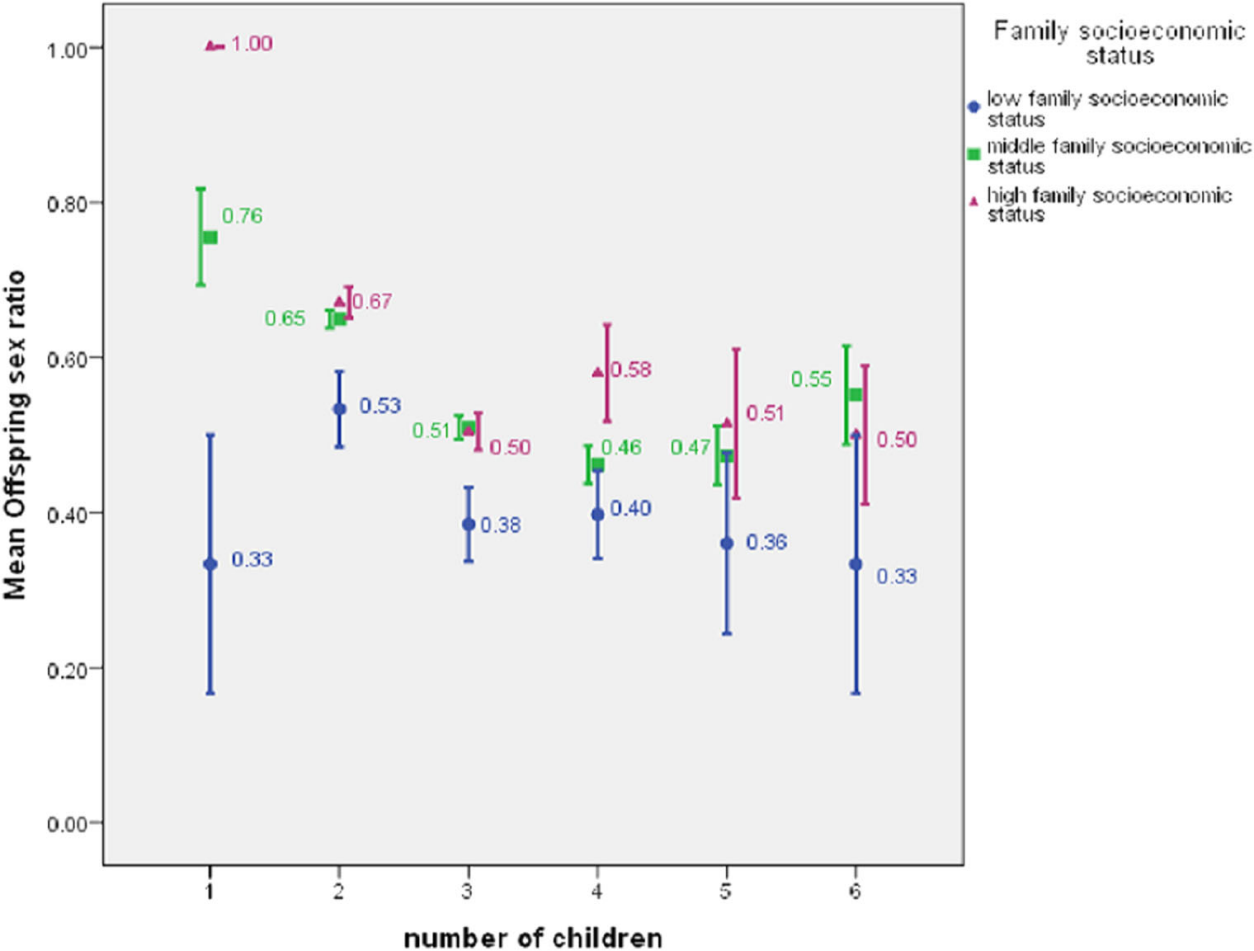
Offspring sex ratio by number of children for family heads with low, middle, or high family socioeconomic status, showing mean ± 1 SE.

For the 1,369 family heads who had children, the maximum number of offspring was six, and the overall mean offspring sex ratio was 0.586. For 104 low-status, 974 middle-status, and 291 high-status family heads, the mean offspring sex ratios were 0.444 (SD = 0.312), 0.589 (SD = 0.278), and 0.624 (SD = 0.261), respectively (Fig. 2). For the six groups within which a family head had 1, 2, 3, 4, 5, or 6 children, the general pattern that status was positively associated with offspring sex ratio was still there (Fig. 3). Low-status family heads had the least offspring sex ratios in all the six groups, while in four out of the six groups, high-status family heads had the highest sex ratios.

Furthermore, the pattern was confirmed by the ordered probit model that controlled such variables as family head’s age and years of education (Table 4). The model also showed that as a family head’s number of children increased, the offspring sex ratio tended to decline. In all the 6 probit models for the separate parity levels (Table 5), the coefficients of the variable family socioeconomic status were positive. If the significance level was set at 0.10, then only the coefficient of family socioeconomic status in the model at parity 6 would not be statistically significant. But in this model, the number of family heads was only 23. For more complete results of the 6 probit models, see Supplemental Information.

**Table 4:** Ordered probit parameter estimates, regression of each family head’s offspring sex ratio on independent variables.

Independent variables	Coef.	SE	*p*	95% Conf.	
				Lower limit	Upper limit
Sex (male = 1)	0.122	0.095	0.202	−0.065	0.309
Age	0.017	0.003	0.000	0.011	0.024
Years of education	−0.025	0.014	0.086	−0.053	0.003
Number of offspring	−0.438	0.036	0.000	−0.510	−0.367
Father’s former class identity	−0.003	0.042	0.948	−0.085	0.079
Family socioeconomic status	0.328	0.057	0.000	0.216	0.440

**Table 5:** Ordered probit parameter estimates for separate parity levels, regressions of each family head’s offspring sex ratio on independent variables.

Parity level	Coef. of family socioeconomic status	SE	*p*	Pseudo R2
Parity 1 (*n* = 71)	1.314	0.433	0.002	0.181
Parity 2 (*n* = 713)	0.267	0.093	0.004	0.013
Parity 3 (*n* = 390)	0.215	0.115	0.062	0.034
Parity 4 (*n* = 130)	0.355	0.179	0.047	0.096
Parity 5 (*n* = 42)	0.616	0.348	0.077	0.055
Parity 6 (*n* = 23)	0.101	0.405	0.803	0.023

The effect size (Spearman *r*_s_) for current family socioeconomic status in predicting offspring sex ratio is 0.113 (*n* = 1,369, *p* = 0.000).

## Discussion and Conclusion

Our study discovers that current family socioeconomic status is positively associated with family head’s father’s socioeconomic status in around 1950 in a Chinese rural population. This conforms to the first TWH assumption in humans; that is, there are relatively stable transfers of socioeconomic status across generations. This is not a surprising finding at all. First, there has been widespread evidence that intergenerational transfers of socioeconomic status to some degree is a normal situation in both developed and developing countries (Breen & Jonsson, 2005; Erikson & Goldthorpe, 1992; Lin & Bian, 1991). Second, a similar intergenerational transmission process took place in Hungary. Similar to China, Hungary experienced the communist takeover in 1949 and the collectivization of agriculture in the 1950s. However, the new peasant entrepreneurs in the early 1980s emerging during market reform tended to be the descendants of those middle and wealthy peasant families before 1949 (Szelényi, 1988). Third, similar studies in rural China on the effect of father’s previous class identity found positive intergenerational correlations of education, family wealth, or overall family socioeconomic status (Luo, Zhao & Weng, 2016; Sato & Li, 2008, 2013).

We have also shown that low-status family heads have more grandchildren through their daughters than their sons, whereas high- or middle-status family heads show the opposite pattern. Since this finding is generally consistent with the direct inference following from the TWH assumptions, it confirms the applicability of the assumptions in the study population. We cannot imagine what other conditions could have led to this result. As the TWH assumptions are met, it is a matter of course that we find what the TWH predicts: as family heads’ status declines, they tend to produce a lower offspring sex ratio. The present study adds to an earlier study by Luo, Zhao & Weng (2016) which also provides support for the TWH in a contemporary rural Chinese context. In addition, both studies discover a significantly inverse relationship between offspring sex ratio and parity, as is consistent with past findings (Chahnazarian, 1988; Lazarus, 2002). That sex ratio tends to decrease as parity increases should be a general pattern in human populations, as a cross-country comparative study by Mace & Jordan (2005) discovered that sex ratio at birth was negatively correlated with fertility rates.

Luo, Zhao & Weng (2016) conducted their study in Shaodong County, Hunan Province (another province of China). It is well known that east coast and big and middle-size cities in China are economically most developed, and a county in China only includes small towns and rural areas within its territory. The gross domestic product (GDP) per capita in Shaodong in 2014 is about 4,500 US dollars by the present USD to RMB exchange rate (Bureau of Statistics of Hunan Province, 2015). Shaodong should probably be counted as an economically moderately developed county in China. In contrast, Shenqiu County of Henan Province, where the current study was conducted, is a National-Level Poor County designated by the Chinese government, meaning that this county is one of the poorest counties in China. In fact, according to the Bureau of Statistics of Henan Province (2015), the GDP per capita in Shenqiu in 2014 is around 3,100 US dollars.

Although both provinces of Hunan and Henan are sometimes considered a part of central China, central China is almost a pure geographical concept. In contrast, the concepts of northern and southern China are often used to indicate two regions very different from each other in geography, culture, and physical traits. The geographical dividing line between northern and southern China is usually thought to be the Yangtze River (Talhelm et al., 2014) or the Huai River–Qin Mountains Line (Fairbank & Reischauer, 1989) to the north of the Yangtze River. No matter which line is applied, undoubtedly Shenqiu of Henan belongs to northern China and Shaodong of Hunan is in southern China. The weather in the south is more suitable for rice cultivation, while other grains such as wheat are more suitable in the north. Accordingly, usually Northerners prefer wheat-based food to rice-based food, while Southerners prefer rice-based food. All these apply to Shenqiu as a northern county and Shaodong as a southern county. Northerners usually are thought to be physically stronger and taller. In fact, studies (Zhang & Huang, 1988; Morgan, 2000) showed that northern children were heavier and taller than their southern peers. Researchers attributed height difference between Northerners and Southerners to genetic as well as non-genetic factors such as diet and climate (Morgan, 2000), but it seems that a definite, well-accepted explanation is still needed. Also, another study (Talhelm et al., 2014) reported that Southerners were more interdependent and holistic-thinking than Northerners. We cannot see any reasonable grounds that the general pattern does not hold for Shenqiu and Shaodong. To conclude, thus far, we have shown that although the two populations in Shaodong and Shenqiu are very different in economic development, culture, and physical traits, the TWH prediction applies to both of them.

Luo, Zhao & Weng (2016) found that even low-status peasants in Shaodong had more sons than daughters among their offspring absolutely, whereas this study shows that low-status peasants in Shenqiu have more daughters than sons. It is probable that this difference is mainly due to the fact that Shenqiu is among the poorest counties in China. Social scientists (Li & Cooney, 1993) often assert that the Chinese as a whole have a deep-rooted, prevalent cultural norm of son preference, further resulting in a severe shortage of girls, while neglecting the internal differentiation associated with socioeconomic status. Therefore, our finding that low-status peasants in one of the poorest regions have more daughters among their offspring seems to show that an evolutionary perspective can bring important insights for traditional social science studies.

Luo, Zhao & Weng (2016) also found that both current family socioeconomic status and father’s former class identity could predict offspring sex ratio, but in this study only current family socioeconomic status has the predictive power. Perhaps an important reason for this difference is that some family heads whose father’s class identity is categorized as something other than poor peasant/landless laborer and who have relatively high offspring sex ratios have moved out to other places such as cities. Another possibility is that the numbers of family heads whose father’s class identity is middle peasant, rich peasant or landlord are very small in this sample.

The TWH represents an evolutionary or ultimate explanation for sex ratio adjustment in relation to parental conditions, but the proximate or mechanistic reasons for the adjustment is in controversy. As far as this study is concerned, some possible physiological mechanism for the adjustment may be related to physiological or psychological stress. Previous studies (Bruckner, Catalano & Ahern, 2010; Ruckstuhl et al., 2010; Catalano, Yorifuji & Kawachi, 2013) discovered that short-term stress was related to low offspring sex ratios. In our case, a lower socioeconomic status and corresponding poor living condition might induce stress reactions more frequently, further leading to a lower offspring sex ratio at birth. In addition, former studies regarding the TWH (Cronk, 1989; Dickemann, 1979) demonstrated that sometimes behavioral mechanisms were an important reason for sex-ratio bias. This should also apply to the population we studied. Researchers often attributed the high sex ratios observed in China in the past decades to traditional son-preference culture and the consequent sex-selective abortion (Ebenstein, 2010) and differential mortality rates (Coale & Banister, 1994). When the logic of the TWH is applied to peasants in Shenqiu, we predict that high- and middle-status peasants are more likely to have a son preference and thus to abort a female fetus or abuse or even discard daughters. To sum up, the biased sex ratios reported by us are possibly due to some combination of physiological and behavioral mechanisms. But we need evidence to confirm this.

From an evolutionary perspective, if the TWH assumptions are met in a population, it is almost unimaginable that the Trivers–Willard effect will not take place. Yet the size of the Trivers–Willard effect is usually thought to be small (Cronk, 2007). This is not surprising, because the world of life seems often, compared to the physical world, to be influenced by many more factors. In our opinion, there are two cases in which the Trivers–Willard effect may be too small to be detected. The first case is involved with small social inequalities. A prerequisite of the TWH is differentiation on socioeconomic status to some extent. If social inequalities in a population are small, then the Trivers–Willard effect probably will be a really very small effect that is difficult to detect. The second case is with the complication of social stratification in industrial societies (Lenski, Nolan & Lenski, 1995; Kerbo, 2009). In this case, when a whole national population is assumed to be a population in a biological sense, we often can only expect very small Trivers–Willard effects, as some researchers (Gelman & Carlin, 2014) did. Consider a national society that comprises many strata based on differentiated occupation, income, prestige, etc. If marriages and social behaviors take place mainly within each stratum, social class, or ethnic group living in a region, perhaps we had better regard this national society as a medley of populations, rather than a population in a biological sense. In fact, studies show that people do tend to marry within their social group (Kalmijn, 1998). Nonetheless, the TWH was conceived by Trivers & Willard (1973) to apply to a single population. Even though such a national society can be roughly regarded as a biological population, the applicability of the TWH in it may be somewhat discounted and requires more accurate measurement. Understanding this may help us further understand why studies failing to provide good support for the TWH seem to tend to be those large-scale studies in industrial national societies (Keller, Nesse & Hofferth, 2001; Koziel & Ulijaszek, 2001). That the whole population in a modern nation sometimes should not be regarded as a population in a biological sense seems to have implications for evolutionary social sciences in general, as evolutionary reasoning often only applies to a biological population.

There are some potential limitations to this study. First, we relied on interviewees’ subjective evaluation to gauge peasants’ family socioeconomic status. If we had found some objective, reliable measure of status, we could have better assessed the validity of the interviewees’ opinions. We once planned to use self-reported income for measuring peasants’ economic status. But later we found that peasants had difficulty reporting accurate income, because they have multiple income sources and they may sell products several times in a year. Also, we once planned to use housing conditions for measuring socioeconomic status, but housing conditions seem to be difficult to quantify. Second, when examining the relationships between socioeconomic status around the period of parental investment and reproduction, this study and some other studies (Hopcroft & Martin, 2014; Nettle & Pollet, 2008; Wallner, Fieder & Seidler, 2012) used current status or status at a certain point in time to make an estimate. This should not be a big issue, because there seems no good reasons to expect that the status of many individuals in such a population as we studied had been changed suddenly in their lifetime. On the other hand, we found in our preliminary fieldwork that reported status during parental investment was vulnerable to recall bias. Third, the interviewees might forget to report some births for some reason, and thus the real sex ratios might be somewhat distorted. In view of the interviewees’ close familiarity with local residents and the large sample size of this study, however, it is difficult to see how this could have modified the pattern of biased sex ratios we report.

## Supplemental Information

10.7717/peerj.3546/supp-1 Supplemental Information 1 TWH raw data.

10.7717/peerj.3546/supp-2 Supplemental Information 2 Supplemental Information.

## Additional Information and Declarations

### Data Availability

The following information was supplied regarding data availability: Luo, Liqun (2017): TWH HENAN DATA. figshare. https://doi.org/10.6084/m9.figshare.4977635.v1
