# Supplementary material for: Socioeconomic status influences sex ratios in a Chinese rural population
Source: PeerJ. 2017 Jun 30;5:e3546. doi: 10.7717/peerj.3546 (PMC5494181; doi:10.7717/peerj.3546)
Supplement: Supplemental Information 2 [file peerj-05-3546-s002.docx]

**Supplemental Information #1.**

**Ordered probit parameter estimates at parity 1, regression of each family head’s offspring sex ratio on independent variables.**

N=71, Pseudo R^2^=0.181.

**Supplemental Information #2.**

**Ordered probit parameter estimates at parity 2, regression of each family head’s offspring sex ratio on independent variables.**

N=713, Pseudo R^2^=0.013.

**Supplemental Information #3.**

**Ordered probit parameter estimates at parity 3, regression of each family head’s offspring sex ratio on independent variables.**

N=390, Pseudo R^2^=0.034.

**Supplemental Information #4.**

**Ordered probit parameter estimates at parity 4, regression of each family head’s offspring sex ratio on independent variables.**

N=130, Pseudo R^2^=0.096.

**Supplemental Information #5.**

**Ordered probit parameter estimates at parity 5, regression of each family head’s offspring sex ratio on independent variables.**

N=42, Pseudo R^2^=0.055.

**Supplemental Information #6.**

**Ordered probit parameter estimates at parity 6, regression of each family head’s offspring sex ratio on independent variables.**

N=23, Pseudo R^2^=0.023.
